# Genetic and Clinical Features of Heterotaxy in a Prenatal Cohort

**DOI:** 10.3389/fgene.2022.818241

**Published:** 2022-04-19

**Authors:** Tong Yi, Hairui Sun, Yuwei Fu, Xiaoyan Hao, Lin Sun, Ye Zhang, Jiancheng Han, Xiaoyan Gu, Xiaowei Liu, Yong Guo, Xin Wang, Xiaoxue Zhou, Siyao Zhang, Qi Yang, Jiaqi Fan, Yihua He

**Affiliations:** ^1^ Beijing Anzhen Hospital, Capital Medical University, Beijing, China; ^2^ Beijing Lab for Cardiovascular PrecisionMedicine, Beijing, China; ^3^ Beijing Advanced Innovation Center for Big Data-based Precision Medicine, Capital Medical University, Beijing, China; ^4^ Key Laboratory of Medical Engineering for Cardiovascular Disease, Ministry of Education, Beijing, China; ^5^ Department of Ultrasound, Peking University International Hospital, Beijing, China

**Keywords:** congenital heart, heterotaxy syndrome, whole exome, CNV (copy number variant), echocardiagraphy

## Abstract

**Objectives:** Some genetic causes of heterotaxy have been identified in a small number of heterotaxy familial cases or animal models. However, knowledge on the genetic causes of heterotaxy in the fetal population remains scarce. Here, we aimed to investigate the clinical characteristics and genetic spectrum of a fetal cohort with heterotaxy.

**Methods:** We retrospectively investigated all fetuses with a prenatal diagnosis of heterotaxy at a single center between October 2015 and November 2020. These cases were studied using the genetic testing data acquired from a combination of copy number variation sequencing (CNV-seq) and whole-exome sequencing (WES), and their clinical phenotypes were also reviewed.

**Result:** A total of 72 fetuses diagnosed with heterotaxy and complete clinical and genetic results were enrolled in our research. Of the 72 fetuses, 18 (25%) and 54 (75%) had left and right isomerism, respectively. Consistent with the results of a previous study, intracardiac anomalies were more severe in patients with right atrial isomerism than in those with left atrial isomerism (LAI) and mainly manifested as atrial situs inversus, bilateral right atrial appendages, abnormal pulmonary venous connection, single ventricles or single atria, and pulmonary stenosis or atresia. In 18 fetuses diagnosed with LAI, the main intracardiac anomalies were bilateral left atrial appendages. Of the 72 fetuses that underwent CNV-seq and WES, 11 (15.3%) had positive genetic results, eight had definitive pathogenic variants, and three had likely pathogenic variants. The diagnostic genetic variant rate identified using WES was 11.1% (8/72), in which primary ciliary dyskinesia (PCD)-associated gene mutations (CCDC40, CCDC114, DNAH5, DNAH11, and ARMC4) accounted for the vast majority (n = 5). Other diagnostic genetic variants, such as KMT2D and FOXC1, have been rarely reported in heterotaxy cases, although they have been verified to play roles in congenital heart disease.

**Conclusion:** Thus, diagnostic genetic variants contributed to a substantial fraction in the etiology of fetal heterotaxy. PCD mutations accounted for approximately 6.9% of heterotaxy cases in our fetal cohort. WES was identified as an effective tool to detect genetic causes prenatally in heterotaxy patients.

## Introduction

Heterotaxy syndrome is a congenital disorder resulting from incorrect establishment of left–right (LR) patterning during embryogenesis. It is a relatively uncommon syndrome, with an incidence rate of approximately 1:7,000–1:5,000 (Lin et al., 2000; Reller et al., 2008). Classic heterotaxy is characterized by abnormally arranged thoracic and visceral organs and a relatively high mortality mainly due to cardiac defects. Owing to the complexity of this disease, there is no consensus regarding the precise nomenclature of heterotaxy and nosological relationships between clinical and phenotypic features of patients with heterotaxy; however, it is generally classified as left or right isomerism. Clinical outcomes are remarkably poorer in patients with right isomerism than in those with left isomerism (Baban et al., 2018). Moreover, despite surgical management, the occurrence of congenital heart defects associated with heterotaxy has a relatively poor survival associated compared to that of patients with similar congenital heart defects but without heterotaxy (Bartz et al., 2006; Kim et al., 2008). Hence, heterotaxy is not only a disease with significant phenotypic heterogeneity but also a disorder with a considerable medical and economic burden associated with it.

The molecular mechanisms of LR patterning have been extensively investigated in animal models, and numerous implicated genes have been identified. However, only a few of them are candidate genes for causing heterotaxy in humans (Ma et al., 2012; Bellchambers and Ware, 2018). In 1997, the zinc finger protein of the cerebellum 3 gene (*ZIC3*) was first identified as the cause of heterotaxy based on familial X-linked pedigrees. However, mutations in *ZIC3* explain only 3–5% of sporadic heterotaxy cases (Bellchambers and Ware, 2018). Similarly, although the Nodal signaling pathway is a conserved and well-established pathway involved in embryonic LR development, point mutations in members of the Nodal pathway are found only in 5–10% of patients with heterotaxy (Dykes, 2014; Grimes and Burdine, 2017; Pelliccia et al., 2017). Currently, known genes account for only approximately 10–20% of sporadic cases (Sutherland and Ware, 2009; Fakhro et al., 2011). As its specific genetic etiology is currently identifiable in only a minority of patients, numerous novel genes and pathways implicated in the pathogeny of this disorder are expected to be discovered in the next years.

During the past decade, copy number variants (CNVs) and single-nucleotide polymorphisms (SNPs) that are associated with heterotaxy have been identified using G-banding and chromosome microarray analysis (Cowan et al., 2016). Moreover, whole-exome sequencing (WES) has been slowly implemented in the clinical setting in recent years as it can detect intragenic variants and lead to a more comprehensive genetic diagnosis (Li et al., 2019). WES enables the rapid analysis of a large number of gene sequences and has permitted the discovery of genes involved in numerous genetic diseases. To date, only a few studies have used WES to uncover the genetic etiology of congenital abnormalities, and most of them have focused on the study of postnatal cases (Lord et al., 2019; Diderich et al., 2021). To the best of our knowledge, no studies have used WES for the prenatal genetic diagnosis of heterotaxy.

In this study, the combination of CNV sequencing (CNV-seq) and WES was used to detect causative genes and mutations associated with heterotaxy in a prenatal cohort. We aimed to determine the characteristics of the genetic variants identified using WES and to evaluate the diagnostic value of WES in patients with heterotaxy. To this end, fetus, mother, and father trios were sequenced simultaneously. The detected variants were then interpreted following the guidelines recommended by the American College of Medical Genetics (ACMG).

## Materials and Methods

### Patient Recruitment

We retrospectively investigated the previously diagnosed cases of fetuses with heterotaxy at the Beijing Anzhen Hospital, Capital Medical University, from October 2015 to November 2020. Clinical data, including gestational age at diagnosis, family history, indications for fetal echocardiography, and pregnancy outcomes, were collected. The available postnatal echocardiograms and autopsy studies were reviewed to confirm the diagnosis of heterotaxy. This study was approved by the research ethics board of Anzhen Hospital, and informed consent was obtained from the parents.

### Echocardiographic Data and Phenotypic Classification

Fetal echocardiography was performed using a segmental approach with standardized anatomical planes and pulse-wave and color Doppler imaging. All cases were diagnosed by two independent investigators with extensive experience in the field of fetal cardiology (Carvalho et al., 2013). Heterotaxy was diagnosed when fetuses exhibited visceral situs ambiguity and complex congenital heart disease (CHD) (Primo et al., 2020).

Right atrial isomerism (RAI) was diagnosed in the presence of characteristic cardiac anomalies, including atrioventricular septal defect, conotruncal lesions, anomalous pulmonary venous return, and atrial appendages on both sides of the body that have the appearance of a morphologically right atrial appendage, and with at least one of the following findings: 1) juxtaposition of the abdominal aorta and inferior vena cava (IVC) or 2) visceral heterotaxy, namely, discordant laterality of the stomach, portal sinus, or gallbladder. Left atrial isomerism (LAI) was suspected in the presence of azygos continuation of the interrupted IVC and at least one of the common features of LAI: 1) cardiac defects, 2) heart block, and 3) visceral heterotaxy. The spleen was also assessed to classify the type of atrial isomerism, namely, asplenia in RAI and polysplenia in LAI.

### CNV-Seq

Fetal samples, including dermal biopsies, umbilical cord blood, and parent blood, were collected. All fetus–mother–father trios underwent CNV-seq and WES. Both CNV-seq and WES were performed using a research-based protocol. CNV-seq was performed as described previously (Kearney et al., 2011; Liu et al., 2015). Briefly, RNA-free high-molecular-weight genomic DNA (gDNA) was isolated from the umbilical cord using a QIAGEN DNA Blood Midi/Mini Kit (QIAGEN GmbH, Hilden, Germany) following the manufacturer’s protocol. The quality and concentration of gDNA from the samples were assessed using a Qubit 2.0 Fluorometer (Thermo Fisher Scientific, Waltham, MA, United States). Approximately 5 million sequencing reads from each sample were mapped to the NCBI human reference genome (hg19/GRCh37) using the Burrows–Wheeler Aligner and allocated to 20 kb sequencing bins with a 5 kb sliding window to achieve a high resolution in identifying CNVs. The expected resolution was 100 kb. The CNV profiles of each chromosome were represented as log2 of the mean sequencing reads of each sequencing bin along the chromosome.

Detected CNVs were evaluated based on a scientific literature review and the following public databases: DECIPHER (https://decipher.sanger.ac.uk/), DGV (http://dgv.tcag.ca/), 1000 Genomes Project (http://www.internationalgenome.org/), OMIM (http://omim.org/), ClinVar (http://www.ncbi.nlm.nih.gov/clinvar), ClinGen (https://www.clinicalgenome.org/), and ISCA CNV (https://www.iscaconsortium.org). Following the ACMG and Genomics standards and guidelines for the interpretation of CNVs, CNVs were classified into three categories: benign, uncertain clinical significance, and pathogenic (Riggs et al., 2021). In this study, we only report pathogenic CNVs.

### WES and Data Analysis

gDNA was extracted from the umbilical cord and parental blood using a QIAGEN DNA Blood Midi/Mini Kit. DNA libraries were prepared using an Agilent liquid capture system (Agilent SureSelect Human All Exon V6) according to the manufacturer’s protocol. The size distribution and concentration of the libraries were determined using an Agilent 2100 Bioanalyzer and quantified using real-time polymerase chain reaction. The DNA library was sequenced on an Illumina Hiseq 4000 or Illumina Novaseq for paired-end 150 bp-long reads according to the manufacturer’s protocol. The mean sequencing coverage on target regions of whole-exome sequencing was 103-fold. Raw image files were processed using bcl2fastq (Illumina) for base calling and generating raw data. Low-quality sequencing reads were filtered using a quality score of 20 (Q20). The average read depths were 103X for each case. The reads were aligned with the NCBI human reference genome (hg19/GRCh37) using the Burrows–Wheeler Aligner. The BAM files were subjected to SNP analysis, duplication marking, indel realignment, and recalibration using GATK and Picard.

### Variant Annotation, Filtering, and Classification

After variant detection, wANNOVAR (http://wannovar.wglab.org/) was used for annotation. Variant frequencies were determined using dbSNP150 (https://www.ncbi.nlm.nih.gov/SNP/), the 1000 Genomes Project, Exome Variant Server (http://evs.gs.washington.edu/EVS), ExAC (http://exac.broadinstitute.org/), and in-house databases. Common SNPs (minor allele frequency > 0.1%) were removed. Nonsynonymous, spliced, frameshift, nonframeshift variants and variants located in splice sites within 10 bp of an exon were prioritized for the study. SIFT (http://sift.jcvi.org), PolyPhen-2 (http://genetics.bwh.harvard.edu/pph2), MutationTaster (http://www.mutationtaster.org), and CADD (http://cadd.gs.washington.edu) were used to predict the pathogenicity of missense variants, whereas Human Splicing Finder (http://www.umd.be/HSF) and MaxEntScan (http://genes.mit.edu/burgelab/maxent/Xmaxentscan_scoreseq.html) were used to evaluate the splicing effects. Moreover, databases such as OMIM, ClinVar, and Human Gene Mutation Database (http://www.hgmd.org) were used to determine variant harmfulness and pathogenicity when appropriate. ACMG variant classification recommendations were utilized for all reported variants (Li et al., 2017). Variants were classified into one of the following five categories: pathogenic, likely pathogenic, of uncertain significance, likely benign, or benign.

### Sanger Sequencing Confirmation

Sanger sequencing was performed to confirm all potentially diagnostic genetic variants identified.

### Statistical Analyses

Categorical variables are presented as frequencies (percentages) and were compared using the Pearson χ^2^ test or Fisher’s exact test. Statistical analyses were performed using SPSS version 23 (SPSS Inc., Chicago, IL, United States). Statistical significance was set at *p* < 0.05.

## Results

### Cohort Characteristics

**FIGURE 1:**
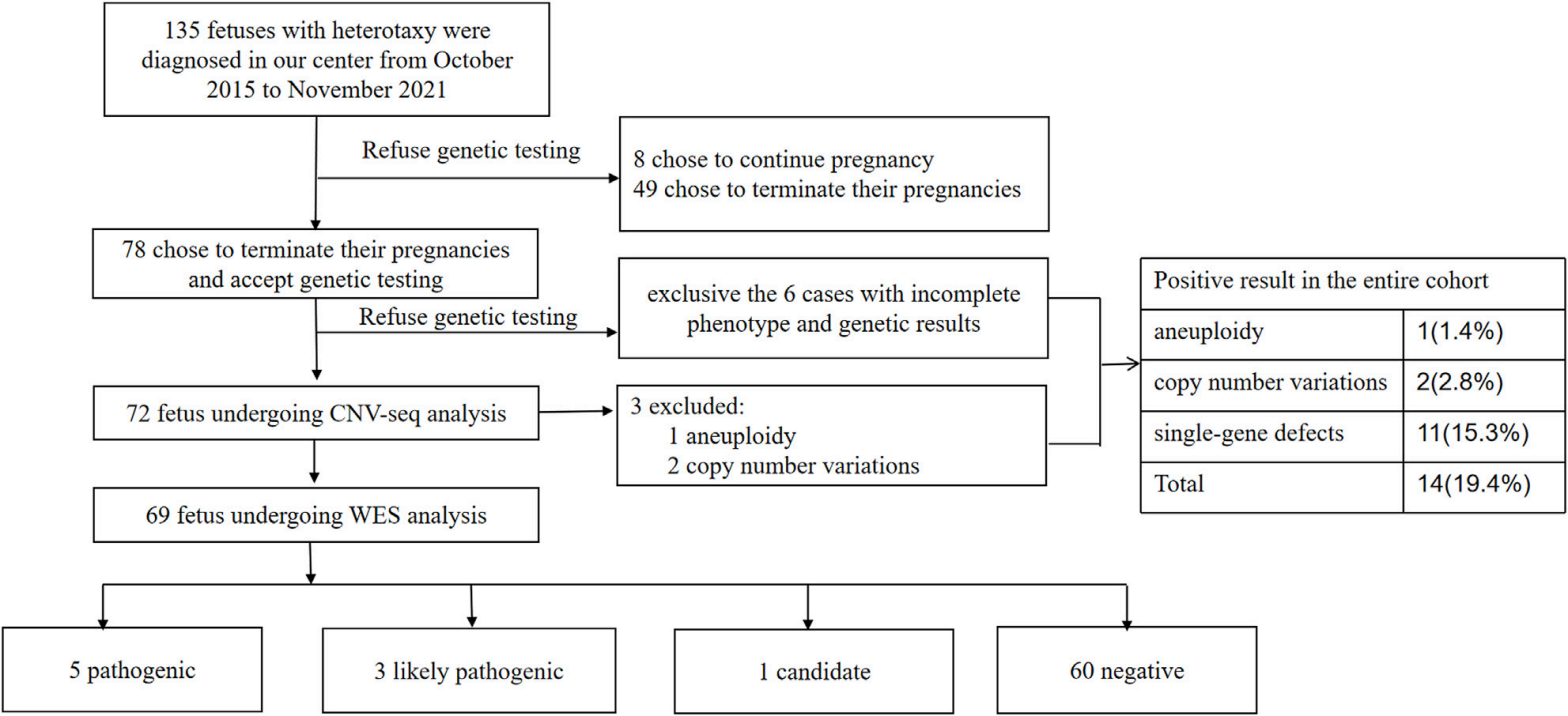
Flowchart summarizing detection of genetic variants by CNV-Seq and WES performed in 72 prenatal samples with heterotaxy. CNV, copy number variation; WES, whole-exome sequencing.

From October 2015 to November 2021, 135 pregnant women found to have fetuses with heterotaxy were screened for inclusion in our study. Among them, eight chose to continue pregnancy without genetic testing and 127 chose to terminate their pregnancy (of which 49 women chose not to undergo genetic testing or to do it at other medical centers or companies). Hence, 78 parents chose to terminate the pregnancy and underwent CNV-seq and WES sequentially at our center. Six cases were excluded because of incomplete imaging or the lack of evidence of heterotaxy. Thus, 72 pregnancies were eligible for the analysis in our study (Figure 1). Autopsy was refused by the parents for 41 fetuses.

All parents were healthy according to their physical examination reports and were nonconsanguineous. The mean maternal age at diagnosis was 28.2 ± 4.11 years, and the median gestational age was 23.74 ± 2.84 weeks. Left isomerism was present in 18 (25%) fetuses, and right isomerism was present in 54 (75%) among all fetuses analyzed (72). The spectrum of cardiovascular abnormalities is shown in Table 1. The main intracardiac anomalies detected among the 54 fetuses with prenatally diagnosed RAI were atrial situs inversus, bilateral right atrial appendages, abnormal pulmonary venous connection, single ventricles or atria, and pulmonary stenosis or atresia. Among the 18 fetuses diagnosed with LAI, the main intracardiac anomaly was the presence of bilateral left atrial appendages. Table 2 summarizes the distribution of visceral abnormalities among patients with left and right isomerism; it also summarizes the visceral abnormalities in patients with RAI. Most RAI patients presented with juxtaposition of the descending aorta and IVC on the same side, aspenia, and bilateral right bronchi. The main extracardiac anomalies in LAI were interrupted IVC, bilateral left bronchi (long), polysplenia, and arrhythmias.

**TABLE 1:** Spectrum of cardiovascular abnormalities in right and left atrial isomerism patients.

	RAI	LAI	Number of patients	P
	N% (95% CI)	N% (95% CI)		
Total number of patients in each column	54	18		
Cardiac abnormality				
Cardiac position				0.035^a^
Levocardia	23 (42.6)	14 (77.7)	37 (51.4)	
Dextrocardia	22 (40.7)	3 (16.7)	25 (34.7)	
Mesocardia	9 (16.7)	1 (5.6)	10 (13.9)	
Atrial arrangement				0.000^b^
Atrial situs inversus	8 (14.8)	1 (5.6)	9 (12.5)	
Bilateral right atrial appendages	29 (53.7)	0	29 (40.3)	
Bilateral left atrial appendage	0	14 (77.8)	14 (19.4)	
No record	17 (31.5)	3 (16.7)	20 (27.8)	
SV/SA	27 (50.0)	3 (16.7)	30 (41.7)	0.013^a^
Non-SV/SA	27 (50.0)	15 (83.3)	42 (58.3)	
AVSD	35 (64.8)	9 (50.0)	44 (61.1)	0.264^a^
Non-AVSD	19 (35.2)	9 (50.0)	28 (38.9)	
Outflow tracts and great vessels				
DORT	21 (38.9)	6 (33.3)	27 (37.5)	0.673^a^
Non-DORT	33 (61.1)	12 (66.7)	45 (62.5)	
TGA	7 (13.0)	2 (11.1)	9 (12.5)	1.000^b^
Non-TGA	47 (87.0)	16 (88.9)	63 ()	
PS or PA	43 (79.6)	8 (44.4)	51 (70.8)	0.004^a^
Non-(PS or PA)	11 (20.4)	10 (55.6)	21 (29.2)	
Truncus arteriosus				0.434^b^
Truncus arteriosus	9 (16.7)	1 (5.6)	10 (86.1)	
Non-truncus arteriosus	45 (83.3)	17 (94.4)	62 (13.9)	
**Aortic arch**				0.380^a^
Left aortic arch	21 (38.9)	8 (44.4)	29 (40.3)	
Right aortic arch	15 (27.8)	7 (38.9)	22 (30.6)	
Unknown	18 (33.3)	3 (16.7)	21 (29.2)	
Venous anomalies				
SVC				0.642^b^
Right SVC	19 (35.2)	8 (44.4)	27 (37.5)	
Left SVC	4 (7.4)	0 ()	4 (5.6)	
Bilateral SVC	20 (37.0)	5 (27.8)	25 (34.7)	
Unknown	11 (20.4)	5 (27.8)	16 (22.2)	
TAPVC/PAPVC				0.028^a^
TAPVC/PAPVC	28 (51.9)	4 (22.2)	32 (44.4)	
Non-TAPVC/PAPVC	26 (48.1)	14 (77.8)	40 (55.6)	

The Chi square or Fisher’s exact test was performed as appropriate; a. Pearson’s Chi-squared test; b. Fisher’s exact test; RAI, right atrial isomerism; LAI, left atrial isomerism; AVSD, atrioventricular septal defect; SV/SA, single ventricle or single atrium; DORT, double-outlet right ventricle; TGA, transposition of great arteries; PS or PA, pulmonary stenosis or atresia; SVC, superior vena cava; TAPVC/PAPVC, anomalous pulmonary venous return.

**TABLE 2:** Spectrum of visceral abnormalities in right and left atrial isomerism.

	RAI	LAI	Total (number of patients, %)	P (b)
IVC				0.000
Interrupted IVC, azygos/hemiazygos vein continuation	1 (1.9)	16 (88.9)	17 (23.6)	
Noninterrupted IVC	53 (98.1)	2 (11.1)	55 (76.4)	
Relationship of IVC and descending aorta				0.053
IVC right of the spine and descending aorta left of the spine	8 (14.8)	0	8 (11.1)	
IVC left of the spine and the descending aorta right of the spine	1 (1.9)	0	1 (1.4)	
IVC and descending aorta same side	22 (40.7)	0	22 (30.6)	
IVC left of the spine and the descending aorta anterior of the spine	6 (11.1)	0	6 (8.3)	
IVC anterior of the spine and the descending aorta left of the spine	0	1 (5.6)	1 (1.4)	
Unknown	16 (29.6)	1 (5.6)	17 (23.6)	
Bronchi				0.000
Bilateral right bronchi (short)	18 (33.3)	0	18 (25)	
Bilateral left bronchi (long)	1 (1.9)	8 (44.4)	9 (12.5)	
Multiple pulmonary lobes	1 (1.9)	0	1 (1.4)	
Unknown	34 (63.0)	10 (55.6)	44 (61.1)	
Spleen				0.000
Polysplenia	1 (1.9)	12 (66.7)	13 (18.1)	
Aspenia	25 (46.3)	0	25 (34.7)	
Single right spleen	8 (14.8)	0	8 (11.1)	
Single left spleen	1 (1.9)	1 (5.6)	2 (2.8)	
Unknown	19 (35.2)	5 (27.8)	24 (33.3)	
Stomach				0.29
Right-sided stomach	28 (51.9)	6 (33.3)	34 (47.2)	
Left-sided stomach	18 (33.3)	9 (50)	27 (37.5)	
Stomach centrally situated	3 (5.6)	0	3 (4.2)	
Unknown	5 (9.3)	3 (16.7)	8 (11.1)	
Liver				0.511
Left-sided liver	24 (44.4)	6 (33.3)	30 (41.7)	
Liver centrally situated	18 (33.3)	9 (50.0)	27 (37.5)	
Right-sided liver	7 (13.0)	3 (16.7)	10 (13.9)	
Unknown	5 (9.3)	0	5 (6.9)	

The Chi square or Fisher’s exact test as appropriate; a. Pearson’s Chi-squared test; b. Fisher’s exact test. Values are presented as numbers (%). RAI, right atrial isomerism; LAI, left atrial isomerism; IVC, inferior vena cava.

### Frequency of the Genetic Variants in Different Presentations of Heterotaxy

Among the 72 cases analyzed, eight had definitive pathogenic variants and three had likely pathogenic variants. The positive detection rate was approximately 15.3% (11/72). The frequencies of the genetic variants in patients with different presentations of heterotaxy are shown in Supplementary Table S1. The positive genetic detection rate for isomerism of the right atrial appendages was significantly higher than that for isomerism of the left atrial appendage (p = 0.04).

### Diagnostic Yield of CNV-Seq

Among the 72 cases for whom CNV-seq analysis was performed, three (16.9%) had a chromosomal abnormality, including one (7.2%) with aneuploidy (21 chromosome trisomy) and two (9.6%) with a *de novo* pathogenic CNV (22q11.2 deletion). The three cases with such chromosomal abnormalities were prospectively excluded from the WES analysis.

### Contribution of Single-Gene Defects

Of the 69 patients for whom WES analysis was performed, 11.6% (8/69) had a positive result, indicating an increased yield of 11.1% (8/72) and an overall diagnostic yield of 15.3% (11/72) in the entire cohort (Table 1). The genotype–phenotype information of the eight cases is shown in Table 3. The ratio of recessive genotypes was 8.3% (6/72), whereas dominant mutations accounted for 4.2% (3/72) of heterotaxy patients. *De novo* mutations accounted for 2.8% (2/72) of patients.

**TABLE 3:** Genotype–phenotype information of patients diagnosed with pathogenic variants, likely pathogenic variants, or variants of uncertain significance.

Gene	Patient ID	Clinical phenotypes	NM	Variants		Parental origin	Zygosity	Variant (novel/reported) (PMID)	Gene-related phenotypes (phenotype MIM number)	Variant class
				cDNA	protein					
ARMC4	21	RAL (AVSD, RVOT, PS, SRS, RSS, LCS)	NM_001290021	c.1454G > A	p.G485D	Mat/Pat	Compound heterozygous	Novel	Ciliary dyskinesia, primary,23,AR (615451)	P
				c.722T > G	p.L241R			Novel		
CCDC114	1	RAL (HLHS, TAPVC, asplenia, RSS, BRB)	NM_144577.3	c.761_768delGCGTCTGG	p.G254Efs*17	Mat/Pat	Compound heterozygous	Novel	Ciliary dyskinesia, primary,20,AR (615067)	P
				c.88G > A	p.R30W			Novel		
DNAH11	2	RAL (AVSD, PA, TAPVC, SVC, asplenia, RSS, LSL)	NM_003777.3	c.3470T > G	p.L1157R	Mat/Pat	Compound heterozygous	32502479; 31040315; 31507630	Ciliary dyskinesia, primary,7,AR (611884)	P
				c.7628G > T	p.C2543F			Novel		
KMT2D	55	RAL (SV, polysplenia, SA, RSS, ICP)	NM_003482	c.6595delT	p.Y2199fs*64	*De novo*	Heterozygous	Novel	Kabuki syndrome 1,AD (147920)	P
STRA6	17	RAL (SV, PA,SVC, TAPVC, asplenia, RSS, LCS)	NM_001199042.1	c.523+5C > T		Mat/Pat	Homozygous	Novel	Microphthalmia syndromic 9,AR (601186)	P
CCDC40	57	RAL (SV, PS, RSS, LSL)	NM_017950.3	c.2552G > A	p.R851Q	Mat/Pat	Compound heterozygous	Novel	Ciliary dyskinesia, primary, 15,AR (613808)	LP
				c.2843_2874de	p.G948fs*69			Novel		
DNAH5	25	RAL (SA, SV, PS, SVC, RSS, LSL)	NM_001369	c.1126G > T	p.A376S	Mat/Pat	Compound heterozygous	Novel	Ciliary dyskinesia, primary,3,AR (608644)	LP
				c.2047C > T	p.R683W			Novel		
FOXC1	43	RAL (LCS, RSS, CAT, SV, AVSD, CAT)	NM_001453	c.1124_1125insCGA	p.G375delinsGD	*De novo*	Heterozygous	Novel	Anterior segment dysgenesis 3,AD (601631) Axenfeld–Rieger syndrome,AD (602482)	LP
FGFR3	70	RAL (PA-VSD, asplenia, BRB, and other extracardiac abnormalities*)	NM_001163213	c.1144G > A	p.G382R	*De novo*	Heterozygous	29080836	Achondroplasia,AD, (100800)	Candidate

Maternal (Mat); Paternal (Pat); Single right spleen (SRS); Right-sided stomach (RSS); Left-sided liver (LSL); Liver centrally situated (LCS); Single right spleen (SRS); Bilateral right bronchi (BRB); Superior vena cava (SVC); Single ventricle (SV); Single atria (SA); Transportation of great arteries (TGA); Atrioventricular septal defect (AVSD); Isolate cleft palate (ICP); Pulmonary atresia (PA); Pulmonary stenosis (PS);.Common arterial trunk (CAT); Double outlet of the right ventricle (DORV); Hypoplastic left heart syndrome (HLHS); Total anomalous pulmonary venous connection (TAPVC); *Extracardiac abnormalities shown in the last paragraph in the conclusion part.

In our cohort, the largest contribution of pathogenic diagnostic genetic variant-related phenotypes was primary ciliary dyskinesia (PCD). We found evidence of recessive mutations (compound heterozygous alleles) in *CCDC40*, *CCDC114*, *DNAH5*, *DNAH11*, and *ARMC4*. All these genes are known to cause PCD (MIM 242650). The remaining likely pathogenic and candidate variants were identified in genes that have not been previously reported to be associated with heterotaxy, including *FOXC1*, *KMT2D*, and *FGFR3*. These genes have been reported to be related to heart development, except for *FGFR3*. The patient with the mutation in *FGFR3* had extracardiac phenotypes of disorders of the cervical vertebrae (C4–7), lymph node cysts of the neck, abnormal appearance of the right ear, defects of the right radius, defects of the right thumb, single umbilical arteries, abnormal running of the portal vein, asplenia, and a double right main bronchus.

## Discussion

To the best of our knowledge, this is the first cohort-based study assessing the contribution of genetic variants to heterotaxy in a fetal population. Most importantly, we identified genetic abnormalities in 11 (15.3%) fetuses. These abnormalities represented aneuploidies, CNVs, and diagnostic genetic variants in 1.4, 2.8, and 11.1% of the cases, respectively. WES combined with CNV-seq, rather than just CNV-seq or targeted panels, which only provide partial information about the genome, led to a dramatic rise in the detection rate of genetic variants associated with heterotaxy in our prenatal cohort. This finding supports the notion that WES is an effective tool for detecting the genetic causes of heterotaxy as it can detect both chromosomal abnormalities and novel candidate genes.

Importantly, the WES results showed a spectrum of gene variants involved in the occurrence of heterotaxy, highlighting the need for implementing the use of this technique in a clinical setting to facilitate perinatal decision making and management when conventional tests such as karyotype testing or microarray analyses are inconclusive. Nearly half of the diagnostic genetic mutations identified in prenatal heterotaxy patients were associated with PCD, which is a recessive heterogeneous disorder of motile cilia that presents with chronic otosinopulmonary disease and organ lateral defects in 50% of the cases and with heterotaxy in 10% of the cases (Knowles et al., 2013). Moreover, a previous study reported that patients with heterotaxy and PCD had a higher prevalence of respiratory symptoms compared to patients with heterotaxy without PCD (Shapiro et al., 2014). In addition, another study has shown that patients with PCD and mutations in *CCDC40* have a more severe disease course with an earlier onset and a higher prevalence of neonatal respiratory distress than PCD patients without such mutations (Davis et al., 2015). Hence, we recommend the use of WES in combination with standard diagnostic testing in patients prenatally diagnosed with heterotaxy to identify the pathogenic genetic variants and predict severity of the disease after birth.

Point mutations in Nodal signaling pathway members, which are known to be associated with laterality defects, were not routinely identified in our cohort. This may be due to the limitations of the screening conditions used in the previous studies. Numerous mutations identified in *Nodal* by previous studies were classified as benign in our research, according to the ACMG guidelines (Mohapatra et al., 2009). Mutations in *ZIC3* are also well-documented to be related to situs abnormalities. In our cohort, we did not find any pathogenic or likely pathogenic variants of *ZIC3*. This may be because *ZIC3* mutations underlie only a minority (3–5%) of sporadic heterotaxy cases (Bellchambers and Ware, 2018). Expanding the size of the cohort used may contribute to test this hypothesis. Finally, in our study, many genes related to PCD encoding components of cilia or the assembly of motile cilia were identified. This result is consistent with previous research showing that ultrastructural dynein arm defects strongly correlate with the development of situs abnormalities (Knowles et al., 2016). Collectively, these data highlight how the use of different research techniques or cohorts reveals differences in the genetic spectrum of heterotaxy. These differences should be considered in the clinical setting when performing molecular diagnostics.

Two genes that had not been previously associated with heterotaxy but have been found to be involved in early cardiac development (*KMT2D* and *FOXC1*) were identified in this study. The sequence alteration in coding exon 31 of *KMT2D* is identified in one family: a heterozygous deletion of one nucleotide, c.6595delT, predicting a frameshift with a premature stop codon at position 2,262 of the protein p.(Y2199fs*64). The variant was not detected in both parents, confirming that the variant is *de novo*. This variant has not yet been reported in public databases, including the Genome Aggregation Database (gnomAD), the Exome Aggregation Consortium (Exac), 1000 Genomes Project (1KGP), and Exome Sequencing Project (ESP6500). *KMT2D* is a major cause for Kabuki syndrome (OMIM:147920), in which CHD is predominantly presented. Nearly 31–58% of the patient had CHD, including a single ventricle with a common atrium, VSD, ASD, ToF, coarctation of aorta, and patent ductus arteriosus (Niikawa et al., 1988; Digilio et al., 2001). For this reason, the mutation was considered as a pathogenic mutation. Another sequence alteration in coding exon 6 of *FOXC1* is identified in one proband: a heterozygous insertion of three nucleotides, c.11241125insCGA, predicting that the amino acid residue aspartic acid was inserted after position 375, p.G375delinsGD (Table 3). This variant has not yet been reported in public databases, including gnomAD, Exac, 1KGP, and ESP6500. The variant was not detected in both parents, confirming that the variant is *de novo*. One *FOXC1*-related phenotype showed in OMIM was Axenfeld–Rieger syndrome, type 3, the features of which included hearing loss, CHD, dental anomalies, developmental delay, and a characteristic facial appearance, central nervous hypoplasia (Maclean et al., 2005). In conclusion, the mutation was considered as a likely pathogenic mutation.

Additionally, we also identified *FGFR3* as a candidate gene. *FGFR3* is known to cause achondroplasia with rhizomelic shortening of the limbs, characteristic facies, exaggerated lumbar lordosis, a limitation of elbow extension, genu varum, and trident hand (Bellus et al., 1995). In our study, the fetus with a *de novo FGFR3* mutation was diagnosed with RA (PA-VSD, right-sided aortic arch accompanied by a mirror branch, bilateral right bronchi, and the absence of the spleen) and achondroplasia (disordered arrangement of cervical vertebrae, missing right radius, abnormal appearance of the right ear, trident right hand, and a higher measurement volume of the lateral ventricle) (Nelson et al., 1988). However, *FGFR3* has not been reported to be the genetic cause of heterotaxy to date and has rarely been shown to be related to cardiac abnormalities, except for one case of *FGFR3*-related craniosynostosis combined with a cleft mitral valve (Agochukwu et al., 2012). Functional experiments have shown that FGFR3 is highly expressed in primary cardiomyocytes (Touchberry et al., 2013) and regulates the intracellular calcium levels and cardiac contractility in cardiac hypertrophy, suggesting that it plays a role in cardiomyocytes or heart development.

Although prenatal diagnosis of heterotaxy has been well studied, the spectrum of underlying gene variants has rarely been reported. Our retrospective cohort study demonstrated the spectrum of cardiac and other associated anomalies in heterotaxy in a prenatal cohort and confirmed previous findings of fetal atrial isomerism. Our results showed a greater incidence of RAI than LAI, which is consistent with other studies in Asian cohorts (Yan et al., 2008). It has been previously reported that patients with RAI have more severe cardiac abnormalities compared with patients with LAI (Buca et al., 2018). We observed that the positive genetic detection rate is 3 times higher in RAI than in LAI, even though the difference is not significant for right and left isomerism (Supplementary Table S1). In the future, it is necessary to evaluate this in larger cohorts. Based on these results, we recommended the use of genetic testing, particularly for fetuses diagnosed with RAI. Additionally, the results of this study indicate that the genetic spectrum of fetal heterotaxy must be evaluated in larger studies.

### Study Limitations

This study had some limitations. First, the sample size of this study was relatively small. Second, subtle dysmorphic features cannot be determined using fetal ultrasound, and some phenotypes, particularly neurodevelopmental disorders, cannot be determined in the prenatal setting.

Another major limitation of our study is the deviation of our cohort. As a matter of fact, we work in a referral center which is famous for diagnosing fetal cardiac abnomalities, and the patients we see normally have complicate malformations which may be difficult for primary doctors to diagnose. Therefore, the abnormality of the fetus heart we see in our center is more complicated compared to other centers. For this reason, our cohort may not represent the clinical characteristic of whole prenatal heterotaxy patients neither the TOP rate under normal conditions.

If we calculate the termination of pregnancy rate in our cohort, we may be shocked by the extremely high rate. This may be partly explained by the deviation of our cohort we mentioned above, and more importantly, it may be caused by two reasons: first, in China, the decision to choose TOP is considered a private matter of the couple (Lou et al., 2018). Second, high-quality, targeted counseling following prenatal diagnosis of CHD was not available in China in previous years. In China, the TOP following prenatal diagnose was 97.4% in Hunan and 84.8% in Beijing (Yang et al., 2009; Xie et al., 2017; Li et al., 2018). For now, high-quality, targeted counseling following the prenatal diagnosis of CHDs was established and disseminated, and this condition may improve in the future.

## Conclusion

Clinical phenotyping and next-generation sequencing of a cohort of fetuses with heterotaxy revealed that 1. diagnostic genetic variants were the main genetic cause of fetal heterotaxy, 2. mutations related to PCD were common in the prenatal heterotaxy cohort, and 3. mutations in genes related to Nodal signaling were rare. In contrast to other studies based on animal models or human case reports, our study was conducted on a cohort of prenatal heterotaxy cases, which provided novel insights into the genetic etiology of heterotaxy syndrome. Our results raise the question of whether the insights obtained from animal models and isolated cases with heterotaxy can be extended to fetal patients, especially when determining the candidate genes to be included in sequencing panels. Our results also demonstrate that WES is a promising method for identifying diagnostic genetic variants and novel candidate genes in heterotaxy patients.

## Data Availability

All datasets generated for this study are included in the article/Supplementary Material.
