# Supplementary material for: Genetic and Clinical Features of Heterotaxy in a Prenatal Cohort
Source: Front Genet. 2022 Apr 19;13:818241. doi: 10.3389/fgene.2022.818241 (PMC9061952; doi:10.3389/fgene.2022.818241)
Supplement: Supplementary file 1 [file Table1.DOCX]

|  | Positive  (Total:11) | Negative  (Total:61) | Positive n/ total n | % (95%CI) | P |
| --- | --- | --- | --- | --- | --- |
| Left/Right atrial isomerism |  |  |  |  | 0.271^b^ |
| LAI | 1 | 17 | 1/18 | 5.56(-5.03-16.14) |  |
| RAI | 10 | 44 | 10/54 | 18.5(8.16-28.88) |  |
| Cardiac abnormality |  |  |  |  | 0.445^a^ |
| Levocardia | 6 | 31 | 6/37 | 16.23(4.34-28.1) |  |
| Dextrocardia | 5 | 20 | 5/25 | 20(4.32-35.68) |  |
| Mesocardia | 0 | 10 | 0/10 | 0 |  |
| Atrial arrangement |  |  |  |  | 0.177^b^ |
| Atrial situs inversus | 0 | 9 | 0/9 | 0 |  |
| Isomerism of right atrial appendages | 7 | 22 | 7/29 | 24.14(8.56-39.7) |  |
| Isomerism of left atrial appendages | 1 | 13 | 1/14 | 7.14(-6.35-20.63) |  |
| Unknown | 3 | 17 | 3/20 |  |  |
| Ventricular arrangement |  |  |  |  | 0.493^a^ |
| Ventricular situs solitus/inversus | 7 | 32 | 7/39 | 17.95(5.9-30.00) |  |
| Single ventricle | 4 | 29 | 4/33 | 12.12(0.99-23.26) |  |
| AVSD |  |  |  |  | 1.000^b^ |
| Atrioventricular septal defect | 7 | 37 | 7/44 | 15.9(5.1-26.72) |  |
| Without atrioventricular septal defect | 4 | 24 | 4/28 | 14.3(1.32-27.25) |  |
| **Outflow tracts and great vessels** |  |  |  |  |  |
| Aortic arch |  |  |  |  | 0.218^b^ |
| Left aortic arch | 5 | 24 | 5/29 | 17.24(3.49-30.99) |  |
| right aortic arch | 1 | 21 | 1/22 | 4.55(-4.16-13.25) |  |
| unknown | 5 | 16 | 5/21 |  |  |
| TGA |  |  |  |  | 0.337^b^ |
| Transpotation of great arteries | 0 | 9 | 0/9 | 0 |  |
| No Transpotation of great arteries | 11 | 52 | 11/63 | 17.46(8.09-26.83) |  |
| DORV |  |  |  |  | 0.736^a^ |
| Double outlet of the right ventricle | 5 | 22 | 5/27 | 18.52(3.87-33.17) |  |
| No double outlet of the right ventricle | 6 | 39 | 6/45 | 13.33(3.40-23.27) |  |
| PA or PS |  |  |  |  | 0.72^b^ |
| Pulmonary stenosis and atresia | 7 | 44 | 7/51 | 13.73(4.28-23.17) |  |
| No pulmonary stenosis and atresia | 4 | 17 | 4/21 | 19.05(2.25-35.84) |  |
| **Venous anomalies** |  |  |  |  |  |
| SVC |  |  |  |  | 1.0^b^ |
| Right SVC | 4 | 23 | 4/27 | 14.81(1.41-28.21) |  |
| Left SVC | 0 | 4 | 0/4 | 12.12(0.99-23.26) |  |
| Bilateral SVC | 4 | 21 | 4/25 | 16(1.63-30.37) |  |
| unknown | 3 | 13 | 3/16 |  |  |
| IVC |  |  |  |  | 0.44^b^ |
| Interrupted IVC | 1 | 16 | 1/17 | 5.88(-5.3-17.07) |  |
| No Interrupted IVC | 10 | 45 | 10/55 | 18.18(7.99-28.38) |  |
| TAPVC |  |  |  |  | 0.097^a^ |
|  | 2 | 30 | 2/32 | 6.25(-2.14-14.63) |  |
|  | 9 | 31 | 9/40 | 22.5(9.56-35.44) |  |
| **Visceral abnormality** |  |  |  |  |  |
| Bronchi |  |  |  |  | 0.146^b^ |
| Bilateral right bronchi (short) | 2 | 16 | 4/18 | 11.11(-3.41-25.63) |  |
| Bilateral left bronchi (long) | 1 | 8 | 1/9 | 11.11(-9.42-31.64) |  |
| normal | 2 | 4 | 2/6 | 33.33(-4.39-71.05) |  |
| Multiple pulmonary lobes | 1 | 0 | 1 | 1(100-100) |  |
| Unknown |  |  |  |  |  |
| Spleen |  |  |  |  | 0.794^b^ |
| Polysplenia | 2 | 11 | 2/13 | 15.39(-4.23-35.00) |  |
| Asplenia | 5 | 20 | 5/25 | 20(4.32-35.68) |  |
| Single right spleen | 2 | 6 | 2/8 | 25(-5.00-55.01) |  |
| Single left spleen | 0 | 2 | 0/2 | 0 |  |
| Unknown | 2 | 22 | 2/24 |  |  |
| Stomach |  |  |  |  | 0.042^b^ |
| Right-sided stomach | 9 | 25 | 9/34 | 26.47(11.64-41.3) |  |
| Left-sided stomach | 1 | 26 | 1/27 | 3.7(-3.42-10.83) |  |
| Central stomach | 1 | 3 | 1/4 | 25(-17.44-67.44) |  |
| Unknown | 0 | 7 | 0/7 |  |  |
| Liver |  |  |  |  | 0.494^b^ |
| Left-sided liver | 4 | 26 | 4/30 | 13.33(1.17-25.50) |  |
| Liver centrally situated | 4 | 23 | 4/27 | 14.81(1.41-28.21) |  |
| Right-sidedl liver | 3 | 7 | 3/10 | 30(1.60-58.40) |  |
| Unknown | 0 | 5 | 0/5 |  |  |

**Supplementary table 1**  The frequency of the genetic variants in different pathology of heterotaxy.

a. Pearson’s Chi-squared test; b. Fisher’s exact test; RAI, right atrial isomerism; LAI, left atrial isomerism; LAI, left atrial isomerism; AVSD, Atrioventricular septal defect; DORT, Double outlet right ventricle; TGA, Transposition of great arteries; PS or PA, Pulmonary stenosis or atresia; SVC, superior vena cava; TAPVC/PAPVC, Anomalous pulmonary venous return; IVC, inferior vena cava.
